# Palliative care specialists’ perceptions concerning referral of haematology patients to their services: findings from a qualitative study

**DOI:** 10.1186/s12904-018-0289-1

**Published:** 2018-02-21

**Authors:** Dorothy McCaughan, Eve Roman, Alexandra G. Smith, Anne C. Garry, Miriam J. Johnson, Russell D. Patmore, Martin R. Howard, Debra A. Howell

**Affiliations:** 1 Epidemiology & Cancer Statistics Group, University of York, York, YO10 5DD UK; 2 Department of Palliative Care, York Hospital, York, YO31 8HE UK; 3 Wolfson Palliative Care Research Centre, University of Hull, Hull, HU6 7RX UK; 4 Queen’s Centre for Oncology and Haematology, Castle Hill Hospital, Cottingham, HU16 5JQ UK; 5 Department of Haematology, York Hospital, York, YO31 8HE UK

**Keywords:** Cancer, Leukaemia, Lymphoma, Myeloma, Haematology, Specialist palliative care, End of life, Hospice, Qualitative

## Abstract

**Background:**

Haematological malignancies (leukaemias, lymphomas and myeloma) are complex cancers that are relatively common, affect all ages and have divergent outcomes. Although the symptom burden of these diseases is comparable to other cancers, patients do not access specialist palliative care (SPC) services as often as those with other cancers. To determine the reasons for this, we asked SPC practitioners about their perspectives regarding the barriers and facilitators influencing haematology patient referrals.

**Methods:**

We conducted a qualitative study, set within the United Kingdom’s (UK’s) Haematological Malignancy Research Network (HMRN: www.hmrn.org), a population-based cohort in the North of England. In-depth, semi-structured interviews were conducted with 20 SPC doctors and nurses working in hospital, community and hospice settings between 2012 and 2014. Interviews were digitally audio-recorded, transcribed and analysed for thematic content using the ‘Framework’ method.

**Results:**

Study participants identified a range of barriers and facilitators influencing the referral of patients with haematological malignancies to SPC services. Barriers included: the characteristics and pathways of haematological malignancies; the close patient/haematology team relationship; lack of role clarity; late end of life discussions and SPC referrals; policy issues; and organisational issues. The main facilitators identified were: establishment of inter-disciplinary working patterns (co-working) and enhanced understanding of roles; timely discussions with patients and early SPC referral; access to information platforms able to support information sharing; and use of indicators to ‘flag’ patients’ needs for SPC. Collaboration between haematology and SPC was perceived as beneficial and desirable, and was said to be increasing over time.

**Conclusions:**

This is the first UK study to explore SPC practitioners’ perceptions concerning haematology patient referrals. Numerous factors were found to influence the likelihood of referral, some of which related to the organisation and delivery of SPC services, so were amenable to change, and others relating to the complex and unique characteristics and pathways of haematological cancers. Further research is needed to assess the extent to which palliative care is provided by haematology doctors and nurses and other generalists and ways in which clinical uncertainty could be used as a trigger, rather than a barrier, to referral.

## Background

Haematological malignancies (leukaemias, lymphomas and myeloma) are generally considered a complex group of diseases that account for around one in ten of all cancers and affect all ages [1]. Characterised by remitting and relapsing trajectories, unpredictable pathways and divergent outcomes, these diseases range from indolent subtypes that progress slowly over many years, to aggressive conditions with poor prognoses. Although some disease subtypes are considered incurable from diagnosis, new and evolving therapies mean that some patients now have relatively normal life spans; yet others continue to have poor survival, despite intensive treatment [2, 3].

Patients with haematological cancers typically experience symptoms as a consequence of both their disease and/or the side effects of treatment. These manifest physically and psychologically, and are comparable in number, severity and distress to those experienced by patients with other cancers [4–7]. Although the importance of good symptom control and end of life care is widely recognised, there is some evidence of unmet needs in settings outside of the United Kingdom (UK) [6, 8, 9].

Specialist palliative care (SPC) is reported to have a positive effect on quality of life for patients with some cancers [10, 11], and early access to these services is recommended alongside disease directed treatment [12, 13]. For haematological malignancies, evidence suggests that palliative care specialists can optimise symptom management; facilitate more effective coping, accepting and planning for patients (and family members) in dealing with prognostic uncertainty; and act as a communication bridge between the haematologist and the patients, particularly in situations where patients do not fully discuss their fears and concerns with the haematology team [14]. Despite this, research from the UK, United States (US) and elsewhere, indicates that patients with these diseases are not referred to SPC and hospice services as often as people with other cancers [15–18]; and those who access hospice care have poorer health at the time of admission and shorter lengths of stay [19, 20]. These factors are often considered to reflect poor quality end of life care [21].

Referral practices are increasingly the focus of international research (based mainly on reviews of patient records, but also some qualitative studies with haematologists), which provides insights into reasons for late or non-referral of patients with haematological malignancies [22–26]. A recent integrative systematic review, for example, highlights reluctance on the part of haematologists to refer to SPC services due to differing treatment goals, prognostic difficulties, and preference of haematology specialists to manage palliative care, as barriers to referral [27]. However, knowledge of the perspectives of SPC clinicians concerning barriers and facilitators to referral is largely absent [20]. Our study, the first of its kind in the UK, was designed to specifically address this deficit, through interviews with SPC doctors and nurses. The aim of the study was to capture data that enhance understanding of factors that promote or prevent the integration of palliative care and haematology services.

## Methods

### Design

A qualitative approach, incorporating use of semi-structured interviews to generate rich narratives, was used to gain insight and understanding of how participants make sense of the topic under investigation [28].

### Setting

The study was located within the UK’s Haematological Malignancy Research Network (HMRN: www.hmrn.org), a population-based cohort registering all patients newly diagnosed with haematological malignancies across 14 hospitals in the Yorkshire and Humber region [1]. HMRN was established in 2004 by researchers and clinicians to provide infrastructure for an on-going programme of work to generate evidence to improve the clinical experiences of haematology patients and their relatives.

### Participants

A combination of purposive and snowball sampling was used to identify SPC doctors and nurses experienced in working with patients with haematological malignancies across the study area. Potential interviewees were contacted by post, email or telephone, and sent an information leaflet, along with an invitation to participate in the study. All of the 20 palliative care clinicians contacted (including 6 SPC doctors and 14 nurses working in primary care, secondary care and hospice settings) agreed to participate in the study.

### Data collection

After obtaining written consent, in-depth, semi-structured interviews (45–90 min) were conducted privately in participants’ workplaces between 2012 and 2014. With permission from the interviewee, these were digitally audio-recorded, transcribed and anonymised. A topic guide (Table 1), developed from existing literature and the study team’s experiences, was used to guide interviews, and data collection continued until no new information was forthcoming [29].

**Table 1:** Topic guide

1. Perspectives on referral of haematology patients to SPC services
2. Barriers and facilitators to SPC referral
3. Factors specific to the primary/secondary care interface
4. Issues specific to haematology compared to other conditions
5. Key changes that could promote collaboration

### Data analysis

**Fig. 1:**
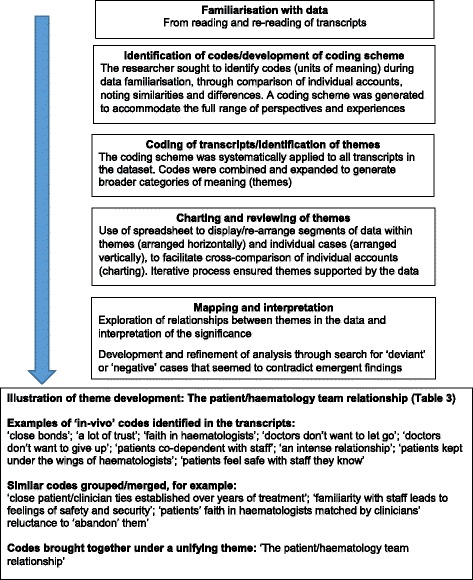
Analysis of data using the ‘Framework’ approach

Transcripts were analysed for thematic content using the ‘Framework’ method [30], whereby a coding scheme and analytical framework were developed, drawing on the topic guide, but incorporating new lines of inquiry identified in the data. Coding and classification was systematic and inductive, involving data familiarisation through reading/re-reading transcripts, coding transcripts and developing analytical categories; followed by identification of common patterns or ‘themes’, interpreted through seeking meaning, salience and connections. Negative or ‘deviant’ cases were actively sought in the data [31], in order to develop and refine the analysis. Data handling and charting, and comparison within and between cases, was facilitated through use of electronic spreadsheets. An overview of the data analysis process is provided in Fig. 1, along with examples illustrating how themes were developed.

Two researchers were involved in the data analysis (DM and DH, both qualified nurses with extensive experience of using qualitative methods in applied health services research settings, and particularly haematology). DM read all of the transcripts and carried out initial identification and compilation of codes, while employing reflective notes and memos. The notes and memos formed the basis of regular discussions (DM and DH), initially to agree and refine codes, and later to discuss emerging themes. Disagreements during discussions offered insights that were useful for refining data coding and interpretation [32]. An experienced researcher (independent of the study) was asked to assess the ‘fit’ of the coding scheme in relation to two interviews [33] and confirmed that the strategy was comprehensive and appropriate [34].

### Ethical considerations

Ethical approval was obtained (Yorkshire and The Humber Research Ethics Committee: 11/YH/0306). Participants were informed they could withdraw from the study at any time, and assurances were given concerning confidentiality and anonymity.

## Results

Study participants identified a range of barriers and facilitators influencing the referral of patients with haematological malignancies to SPC services. Barriers included: the characteristics and pathways of haematological malignancies; the patient/haematology team relationship; lack of role clarity; late end of life discussions and SPC referrals; policy issues; and organisational issues. The main facilitators identified were: establishment of inter-disciplinary working patterns (co-working) and role clarity; timely discussions with patients; access to information platforms able to support information sharing; and use of indicators to ‘flag’ patients’ needs for SPC. Each of these is examined below, with illustrative quotes:

## Perceived Barriers

### **Characteristics and pathways of haematological malignancies** (Table 2)

Interviewees said that the most significant barrier to haematology patients accessing SPC services was the unpredictable trajectories and prognoses associated with the diseases. One commonly cited example of this was the propensity for unexpected deterioration and rapid death, often when patients were being treated with intensive chemotherapy, which resulted in insufficient time to instigate an SPC referral.

**Table 2:** Characteristics and pathways of haematological malignancies

Uncertainty and unpredictability *‘generally, oncology patients follow a kind of downwards steady decline…whereas haematology patients just peak and trough, so it’s hard to predict what somebody is going to do’* (SPC nurse 7) *‘I don’t think it’s quite as easy to predict a lot of the trajectories as it is for some of the other malignancies…it’s not that simple for the haematologists’* (SPC doctor 3)
Lack of clear transition points and treatment late in the pathway *‘other cancers, the oncologists will say “actually there’s no further treatment we can offer at the moment because the side effects are going to outweigh all the benefits now”…they will then discharge them into the community, with the GP. Patients with haematological malignancies tend to get treated and treated and treated…’* (SPC nurse 5) *‘haematologists give more aggressive treatments and do treat up until late into a disease stage, but I think there just is this idea of potentially more to gain’* (SPC doctor 4) *‘even when they’re [patients] on the brink of really being extremely poorly, some weird and wonderful medicines can actually bring them back’* (SPC doctor 3)
Patients’ desire to pursue treatment *‘there was a patient who, even with having a really detailed conversation with a consultant and his family about the poor, poor prognostic outcome of maybe fourth line chemotherapy…the patient wanted to, to take it’* (SPC nurse 14)

Another frequently cited example related to the uncertainty associated with the remitting/relapsing pathways that characterise indolent blood cancers. These often involve patients receiving intermittent life-prolonging treatments (e.g. chemotherapy) and supportive care (e.g. transfusions) over long periods of time, and often close to the time of death, for disease control and to maximise quality of life, with deterioration tending to occur gradually.

Whilst such ongoing therapy was perceived as overly ‘*aggressive’* by some, and a significant barrier to timely SPC referral, others also described how salvage chemotherapy, delivered late in the pathway, could be given due to the possibility of cure or remission, with haematologists weighing up the feasibility of a *‘last ditch treatment (to) pull a patient back from the brink’.* Ongoing treatment was also often said to be desired by patients themselves, even in the face of diminishing response.

Collectively, the propensity for rapid deterioration, remitting and relapsing pathways and treatment close to death, resulted in indistinct transitions in the objectives of care (curative or palliative). This was perceived as a major difference between the pathways of haematology patients and those with other cancers, the latter being more predictable, with clearly demarcated junctures (e.g. recognition that curative treatment had failed), at which time care is often *‘handed over*’ from oncology to palliative care specialists.

### **The patient/haematology team relationship** (Table 3)

Participants described the close bonds that often exist between patients and haematology staff, resulting from frequent and/or prolonged contact from diagnosis onwards, which were said to engender high levels of patient trust in the clinicians’ expertise. While some interviewees perceived this positively, others considered it a potential barrier to SPC referral, due to patients having continuing *‘faith’* in *‘their’* haematologist to *‘come up with’* a cure, or at least life-prolonging treatment. Haematologists were considered reluctant to *‘let go’* of patients (e.g. to SPC staff/services), while active treatment was on-going, and were thought to derive satisfaction from caring for patients from diagnosis until death. These close links were contrasted sharply with patients’ (and haematologists’) lack of familiarity with community-based clinicians, including GPs, district nurses and palliative care nurses.

**Table 3:** The patient/haematology team relationship

Close bonds and trust, and holistic care *‘they’ve [patients] got this relationship that’s gone on for years and years with the [haematology] team and they know them well and feel safe and secure with them’* (SPC nurse 3) *‘[haematologists] develop really close working relationships with these patients, get to know them really well, so it doesn’t always seem easy or appropriate to hand them over to palliative care’* (SPC doctor 3) *‘having built up that relationship over years there is the not wanting to let go…not wanting to give up, there is the strong “let’s just try something else” on the part of the clinicians and patients’* (SPC nurse 10) ‘*they [patients] become very co-dependent with the staff on the ward and the other way round, there is a very close personal relationship… and [haematologists have] that satisfaction of providing all of the care, yeah, holistic care, they’ve done everything for the patient, really.’* (SPC nurse 12)
Contact with community services *‘the majority of haematology patients tend to go back to the ward for any care really. That seems to be their first port of call, rather than accessing community services…they feel the haematology professionals understand their condition more than the generalists in the community’* (SPC nurse 1)

### **Lack of role clarity** (Table 4)

Some SPC clinicians believed haematology staff did not always completely understand their role, perceiving it mainly in relation to symptom control, while not always fully recognising the wider remit, including the provision of psychological and emotional support to patients and family members. This was considered a barrier to patients’ accessing services, and was attributed to teams and individuals working in isolation, with little face-to-face contact, a situation particularly apparent with respect to contact between members of the haematology team and community healthcare providers. Referrals were considered to occur less frequently from haematology compared to other specialities and joint working patterns were not normalised, with pressures on staffing levels sometimes leading to existing co-working practices being curtailed.

**Table 4:** Lack of role clarity

Understanding of role(s) and relationship building *‘it’s understanding one another’s roles, it’s working together. When you don’t get an opportunity to work together often, you don’t build trust and understanding’* (SPC nurse 1)
Limited opportunities for haematologists and community palliative care staff to build relationships *‘it’s not a face-to-face relationship [with haematologists] by us being removed in the hospice and in the community’* (SPC doctor 6)
Dichotomised thinking: *either* active treatment *or* palliative care *‘I think in people’s minds still there is that dichotomy that you’re either having active treatment or you’re having palliative treatment’* (SPC doctor 6)
Negative patient perceptions of hospice services *‘“Gosh, the Macmillan nurse is here, that means I’m going to die…”’* (SPC nurse 14)

Interviewees described some haematologists as taking an *‘either/or’* approach to patient care, representing a form of *‘dichotomised thinking’*, with two sequential stages in the illness trajectory, where active treatment predominates during the first stage, and palliative care in the second. This approach was thought to be associated with the view that palliative care was principally terminal or end of life care, and was said to delay patient access to SPC services.

Importantly, the degree to which patients with haematological malignancies required SPC input was said to be unclear, and it was suggested that end of life care could, in some instances, be adequately met by members of the haematology team, particularly with input from clinical nurse specialists. The extent to which this occurred was uncertain though, and participants noted that specialist nurses, being usually located in busy clinical environments, could have limited time to spend with individual patients and their families.

Additionally, patients were said to lack awareness of the palliative care services that could be available to them, and to hold negative views towards resources such as hospice care *(‘somewhere you go to die’*).

### **Late end of life discussions and SPC referrals** (Table 5)

Introducing discussions with patients about likely treatment and prognostic outcomes was said to be complicated by the difficulties haematologists face in identifying the *‘right’* time to begin conversations (due to prognostic uncertainty); reluctance to broach the subject until all treatment options had been exhausted; and trying to avoid the feeling of *‘giving up’* on the patient*.* Individual haematologists’ skills and confidence in conducting discussions were perceived as variable, and the nature and extent to which haematology specialist nurses are involved in discussions was unclear to interviewees. Patients were also sometimes said to be reluctant to discuss end of life issues, often due to what were considered unrealistic expectations about treatment outcomes.

**Table 5:** Late end of life discussions and SPC referrals

Difficult discussions and having the skills/confidence to initiate them *‘“this chemotherapy has done nothing for you, and actually you are dying”, that must be a really difficult conversation to have…so I think that conversation is sometimes put off’* (SPC nurse 11) *‘I think the [haematology] consultants struggle sometimes…you don’t want to feel you are giving up on them [patients] some are better than others in having discussions with patients’* (SPC nurse 14) *‘if those conversations haven’t been had… then you can’t even begin to start to have the next conversations about preparing for dying’* (SPC nurse 4) *‘with a lot of haematological malignancies a percentage of people can be cured…so [end of life care is] not necessarily something you want to ask at the very beginning when you’re embarking on what might be curative treatment, so it’s quite difficult picking the right time to have those conversations’* (SPC doctor 2)
Timing of SPC referral *‘the acute leukaemias… it’s [referral] very, very end stage, once the marrow is completely failed…the ones we get nearest to are the people with myeloma, where they tend to have lots of bone pain...so we tend to get involved a bit earlier there…’* (SPC doctor 1) *‘by the time [of] the referral, he was imminently dying…that is really, really difficult for us because you don’t have time to build up a relationship, that trust… you often get involved in the very, very end stage. If we could get earlier [referral], build those relationships up so we can explain to patients and their families that we are able to support people at home, to put packages of care to support them, so that they build trust, so they know what contact numbers [to ring], so they don’t have to ring the haematology ward’* (SPC nurse 1)
Patient reluctance to engage in conversations about end of life *‘some haematology patients choose not to know there is no more treatment that they can have… they make a choice not to have this conversation’* (SPC nurse 11)

Interviewees said that SPC referrals were often made late in the pathway, after all treatment options had failed, and when the patient was close to death. Myeloma patients with intractable pain were the recognized exception, many of whom were said to be referred soon after diagnosis. Late referrals were said to cause difficulties, as patients could already be severely ill and relatives/carers highly distressed at this time. Such situations were said to leave SPC staff with little time to establish trust and rapport, elicit end of life preferences and coordinate care before the patient died. Younger patients in particular were described as disinclined to consider SPC referral while receiving treatment, even in the face of diminishing options.

### **Policy issues** (Table 6)

National and local policies were perceived as potentially impeding early access to SPC services for patients with haematological malignancies, with participants citing how specific criteria (such as the presence of complex symptoms, irresolvable by the haematology team) were often necessary before referral was considered justified. Such criteria were cited as inappropriate for some patients with haematological cancers, as they negated referral of individuals without such difficulties, but who could deteriorate unexpectedly and rapidly, and require input at this time. In such circumstances, patients are likely to be disadvantaged as, due to the limited time available before death, they may not accrue the maximum benefits of SPC input. In this context, many interviewees considered the unpredictable nature of haematological cancers sufficient of itself to constitute justifiable referral criteria.

**Table 6:** Policy issues

Palliative care referral criteria *‘there is strict criteria for referral to specialist palliative care in the community…she didn’t meet the criteria’* (SPC nurse 9)
Provision of blood products in hospice settings *‘some hospices will give blood products…we try and avoid giving them out at weekends and during out of hours… every hospice I’ve worked at has had a policy… that when you’re giving blood products you actually need a doctor on site, in case of a reaction’* (SPC doctor 4) *‘the hospice is very much, much more able now to transfuse patients, even with platelets which is much more helpful’* (SPC nurse 14)

Administration of life prolonging therapies (e.g. blood product transfusions and antibiotics), on which many patients depend, was said to conflict with some hospices’ admission criteria, although it was reported that this was changing in some places. Improvements in the willingness of these facilities to administer supportive therapies was considered important if access to hospice services was to be maximised.

### **Organisational issues** (Table 7)

#### ‘Fast track’ discharges

Short notice or ‘fast track’ hospital discharges, where patients were expected to die rapidly and wanted to be transferred home, were considered particularly common in patients with haematological malignancies. Such situations were said to pose significant challenges to the timely co-ordination of multi-agency, community-based nursing and palliative care services, and organisation of the equipment required to facilitate the patient’s return home. Most participants commented that this situation was further complicated because General Practitioners (GPs) had often *‘lost contact’* with patients (who were managed largely in the hospital setting), so could be unaware of their changing prognosis. Participants underscored the importance of GPs receiving as much notice as possible concerning impending patient discharge.

**Table 7:** Organisational Issues

‘Fast-track’ discharge home or to the hospice *‘in haematology patients…can you get people home quick in a very fast changing situation? If you’ve got lung cancer and somebody thinks they’re starting to die, you often have a window to get people home, whereas haematology… you might have a few hours’* (SPC doctor 6) *‘sometimes you can get a [hospice] bed the next day, sometimes you can wait two weeks or more’* (SPC doctor 6)
Lack of GP involvement and impact on the provision of end of life care *‘some GPs, they have haematology patients and they are diagnosed and they don’t see them for two years, three years and then suddenly there is no more treatment for them, they’re discharged home and they’ve [GPs] kind been out of the loop’* (SPC nurse 8) *‘if you’ve got to frequently come to hospital and see the doctors, you don’t tend to find that they also frequently see their GP…so [patients] don’t have the same relationships with the community support staff as some other patients’* (SPC doctor 2)
Access to shared records *‘we’d only get the paper records from haematology anyway…so we don’t really know if any advanced planning [had taken place]…so it’s a big void there’* (SPC nurse 1)

SPC input was also said to be limited by lack of available hospice beds at short notice and variations in the availability of community palliative care services, which were described as *‘patchy’* in some areas, with *‘round the clock’* care being particularly difficult to access.

#### Information platforms

Existing technology was said to limit the sharing of information between disciplines (e.g. palliative care and haematology) and settings (e.g. secondary and primary care). SPC clinicians said that lack of a unified electronic record meant that they were often unable to access details of conversations about prognosis, treatment cessation and end of life care that had taken place between haematology staff and their patients and relatives, requiring them to re-initiate such discussions. Incomplete access to individual patient information was said to potentially impede *‘joined up’* approaches to care, deter the *‘flagging up’* of patients approaching end of life, prevent early identification of those likely to require SPC input, and preclude preferred place of care/death being achieved. Conversely, participants suggested that access to a single record could lead to broader use of end of life tools, such as the Gold Standard Framework.

## Perceived Facilitators

### **Interdisciplinary working patterns (co-working)** (Table 8)

Co-working was said to facilitate early access to palliative care specialist input, which could be delivered alongside the haematology team either continuously (*‘in tandem’*, *‘in parallel’*) or intermittently (where clinicians *‘dip in and out’*). The presence of palliative care specialists in haematology ward rounds, during multidisciplinary team meetings and in clinics was viewed as a time-efficient way for clinicians to contribute to haematology patient care, whilst also providing the opportunity to share expertise. The benefits of co-working were said to be: joint assessment of patients’ needs; opportunities to deliver specific interventions in response to changes; and increased time to build rapport with patients and relatives, consider quality of life issues, explore end of life preferences and participate in advanced planning. Co-working was also said to promote visibility and enhance understanding of roles, factors interviewees considered important in raising their profile and increasing referrals. Co-location of haematology and palliative services was said to foster closer working through regular informal opportunities for inter-professional communication. There was general consensus among the palliative care specialists that their collaboration with haematology was beneficial and desirable, and that it had increased in recent years.

**Table 8:** Interdisciplinary working patterns (co-working)

Working ‘in tandem’ or intermittently *‘getting the palliative care professionals involved quite early, um, so that you’ve got this nice long overlap between the haematology…and the palliative care input so that eventually when somebody is discharged or isn’t being followed up as regularly by the haematologists, they know they are supported by the palliative care team already.’ (SPC doctor 4)* *‘I think we can be involved…and then the patient changes, deteriorates, so we go back in, we sort it out, we come out again…so we have sort of several episodes of care within that journey of care, dependent on what their needs are’ (SPC nurse 5)*
Co-working and visibility *‘the palliative care team attend weekly MDTs, so communication is very good’* (SPC nurse 3) *‘working in partnership with the [haematologists] helps… I think the ward rounds, being that visible person in the ward round, where you can pre-empt some things and then prompt them’* (SPC nurse 5*)* *‘our relationship is really good…we couldn’t go for a period to the ward rounds and referral started to drop off…it’s about our visibility…if we’re there it reminds them that actually they can ask our advice and referrals go up, when we’ve got a high profile, referrals go up’* (SPC nurse 13) *‘the (haematology) consultants here, I have a very good working relationship with them, and I know I can pick up the phone and say, “I’ve seen your patient on the ward today and they’ve expressed these concerns”’* (SPC nurse 14) *‘now it’s a big thing, because referring on to palliative care means you’re [patient] going to be dead in two days… whereas if you’re seen and perceived to be working much closer together, as an integrated team involved from earlier on… then it’s no big deal…’ (SPC doctor 1)*
Co-location *‘we’re quite fortunate in that most of the haematologists are always down this corridor so quite often they will actually pop into our office and talk to us about patients…as well as the ward round, there is that informal opportunity for talking about patients’* (SPC nurse 13)
Growing collaboration between palliative care and haematology specialists *‘I have to say, in the past 10 years, communication [between teams] has improved quite significantly…so that’s obviously been a change for the better’* (SPC nurse 14) ‘*we’ve [SPC nurses] got very close relationships with the haematology wards…there’s a group of us who cover those wards so we’ve built up relationships and they know who we are and they refer to us directly’* (SPC nurse 14) *‘the more time we spend with the haematologists and the more relationships that we build up, the more patients of theirs they refer to us that we help, then the better the relationship gets. It’s all about relationship building. We’re learning about their specialty as much as they’re learning about us and it’s just about shared understanding I think…we have a joint clinic once a week…influencing decision making patient by patient…you’re seen as more as part of the team’* (SPC doctor 6)

### **Timely discussions with patients and early SPC referral** (Table 9)

Early initiation of honest, frank conversations about treatment and prognosis was viewed as a pre-requisite for patient engagement in end of life discussions, advance planning and SPC referral. Participants suggested that early introduction of SPC clinicians as integral members of the care team facilitates a more positive view amongst patients of their increasing involvement towards the end of life, and provides patients (and their family members) with increased opportunities for support (e.g. emotional and psychological support, as well as more time to explore quality of life issues and end of life preferences).

**Table 9:** Timely discussions with patients and early SPC referral

Early initiation of honest, frank conversations *‘I think it’s about being honest with the patient… where they are with the disease… what treatment, management or supportive options are available to them, a little bit earlier’* (SPC nurse 5)
Benefits of early SPC referral *‘in the UK… there is this overlap… and the diagram of the model of care really has palliative care involved almost from the outset… of an ultimately untreatable condition, and increasingly involved towards the end. And certainly.. I think that is something that needs to be adopted, because there is nothing about the active management of a malignant disease that stops palliative care teams getting involved’* (SPC doctor 4)

### **Access to information platforms able to support information sharing** (Table 10)

Shared access to information platforms used by haematologists and other health care professionals (e.g. GPs) was viewed by SPC clinicians as facilitative of patient referral, through improved communication across the primary/secondary interface. It was also said to speed up patient access to available palliative care services.

**Table 10:** Access to information platforms able to support information sharing

*‘we share records with a lot of the GPs and have access to the hospital letters there, so all that information is available…it really helps, you can access what the last things the haematologists have said were…I see this as in a state of flux, something that is improving and something that is very key’* (SPC doctor 4)	

### **Use of indicators to ‘flag’ patients’ needs for SPC** (Table 11)

A means of identifying or ‘flagging’ individuals who might soon require end of life care was highlighted as a potential facilitator to the appropriate timing of SPC referral. Use of a ‘traffic light’ system or ‘Gold Standards’ approach was suggested, along with the development of ‘indicators’ or ‘triggers’, based on diminishing response to treatment, which would signal when an SPC referral should be considered.

**Table 11:** Use of indicators to ‘flag’ patients’ needs for SPC

‘Flagging up’ patients’ needs for SPC *‘the palliative care register, they have the traffic light system…green is that [patients] are ticking along very nicely, and they’re doing alright, amber is that they’re rather slightly deteriorating but, you know, could go either way, and red is that they are deteriorating’* (SPC doctor 2) *“we think this patient is in their last year” and “we think they should go on your Gold Standards (Framework), so at least we would be flagging up to the GP that this is the time to start considering these things”* (SPC nurse 4)
Identification of ‘triggers’ signifying patients could benefit from SPC referral *‘if the patient's response [to transfusions] is either not as good, or not lasting as long, those should be the triggers that the disease is changing, they need to start to broach the idea of palliative input…and to think about involving the GP’* (SPC nurse 6)

## Discussion

This is the first UK study to explore SPC doctors’ and nurses’ perceptions about the referral of patients with haematological cancers to their services. Our findings overlap those arising from research conducted with haematologists [22–24], thus confirming and complementing these studies’ results. New insights we offer include SPC clinicians’ perspectives that their role is not always well understood by haematologists, as well as their perceptions of the importance of co-location of services in promoting and enhancing role clarity and closer working patterns. Improvements such as shared access to information platforms across the specialist disciplines and different care settings were described by study SPC clinicians as fundamentally necessary to enhance patient referral and integrated care delivery. The need for indicators or ‘triggers’ to promote early referral of haematological patients identified in our study has previously been reported in research from the US [26]; our UK findings reinforce these earlier reports, signalling widespread recognition of the requirement for further developments in this area.

Collaboration between disciplines was perceived as beneficial and desirable, though was said to occur less frequently than for patients with other cancers, with a range of issues influencing the likelihood of referral. Barriers largely related to the unique characteristics and pathways of haematological malignancies. Uncertainty (due to fluctuating trajectories, sudden deterioration and death, “last ditch” attempts at salvage, and indistinct transitions) was a significant barrier. Also important were the close connections between patients and the haematology team; late end of life discussions and SPC referrals; organisational issues (such as distant relationships with primary care and specialist palliative care practitioners and limitations to information platforms); lack of role clarity (perceived as “either/or”, curative or palliative) approaches to treatment; and UK policy governing access to SPC services. Facilitators included early referral to SPC services or co-working between disciplines from diagnosis; mutual understanding of roles; timely, frank discussions about prognosis and treatment cessation along with early SPC referral; access to IT platforms able to support information sharing; and the use of indicators to ‘flag’ patients requiring SPC input.

Current UK policy on commissioning palliative care services distinguishes ‘core’ (generalist) from ‘specialist’ providers [35]; in our study, the former comprise haematology staff, GPs and district nurses, and the latter SPC practitioners in hospital, community or hospice settings. The hospital palliative care specialists we interviewed described how the end of life needs of haematology patients may be met by the haematology team, and particularly the clinical nurse specialists. Although we found the extent to which this occurred unclear, another UK study has reported that haematologists considered the delivery of ‘generalist’ palliative care integral to their role, with specialist referrals being made only when they were unable to meet patients’ needs themselves [23]. Without knowledge of such patterns in care, however, it is difficult to determine whether perceptions of fewer SPC referrals reflect unmet need, or whether these needs are simply being met by others. In the UK, there is a lack of clarity around this issue; and until more is known, it is difficult to determine and quantify the extent to which specialist interventions are required.

Whilst unclear transitions and difficulties in prognostication were considered significant barriers to the timeliness of end of life discussions and palliative care referrals, many interviewees considered such characteristics should, in fact, act as triggers for advance planning. Interestingly, this has also been suggested in the context of chronic disease such as advanced heart failure, where similar complexities are reported [36, 37]. Such uncertainties were said to be a major difference between haematological malignancy pathways and those of other cancers. This was undoubtedly linked to the characteristics and pathways of the former diseases, which do not generally adhere to the traditional dichotomy of ‘curative’ and ‘palliative’ phases, with distinct delineation between the two states. Haematology subtypes may, for example, be incurable from diagnosis but manageable with intermittent or continuous chemotherapy, given with ‘life-prolonging’ intent (often late in the pathways) for disease control and to improve quality of life; or may be curable but result in rapid, unexpected death (without transition) due to haemorrhage or sepsis.

Where SPC input was warranted, late referrals were found to be challenging, particularly if the patient’s preference was to die at home, and care systems and equipment were required at short notice to facilitate this, but contact with the primary care team had been lost. The use of indicators or triggers to ‘flag up’ patients nearing the end of life and possibly requiring SPC referral was suggested as a means by which to address these issues, and the use of such systems, including dynamic prognostic tools, has been advocated by US oncologists [26]. Such interventions may, however, have little value in the context of sudden and unexpected deterioration, with the lack of key prognostic indicators, particularly for patients being treated less aggressively or with palliative intent, being a further limitation [38].

Co-working with the haematology team (from diagnosis; concurrently or intermittently; regardless of symptoms or prognosis), or early palliative care referral, were suggested as models by which late referrals could potentially be ameliorated. Such ‘upstream’ integration, which negates the need to identify the end of life phase, has been suggested by others [14, 39–42], including the World Health Organisation [43], who state that “*palliative care is applicable early in the course of an illness, in conjunction with other therapies that are intended to prolong life*”. This would enable patients receiving treatment with curative intent, who may deteriorate suddenly, to begin to forge a relationship with the SPC team prior to their input being required. Other ‘best practice’ models supporting integration of palliative care into haematology settings also suggest early patient referral, as well as a collaborative multi-disciplinary approach to care [44]. Such models appear, however, to be at odds with current UK commissioning guidance, which recommends that ‘specialist’ provision should be restricted to people with ‘*unresolved complex needs that cannot be met by the capability of the current team*’ [35]. Limitations may also be imposed by the additional resources (e.g. adequate numbers of SPC staff) likely to be required to meet this extended role and inconsistencies in hospice policies regarding the administration of life-supporting and prolonging therapies, such as transfusions, on which many patients with haematological malignancies depend [23].

Inter-disciplinary electronic communication was considered crucially important by participants, yet was restricted by the IT systems available to practitioners within their workplace, some of which facilitated information sharing whilst others did not. A recent UK report concluded that failings in communication within and between palliative care teams in primary and secondary care was a major contributor to inadequate service provision [45]. Within England, a novel unified clinical record (the Electronic Palliative Care Coordination Systems - EPaCCS) is currently being implemented to address this deficit. This process has not been without challenge [46] and the impact EPaCCS has on future service delivery will be of interest.

The importance of mutual understanding of roles has been identified in a UK interview study that explored haematologists’ views about collaboration with palliative care services, along with the need for consistent and flexible service provision [23]. In our own study, co-location of services was considered an important means of promoting more integrated working patterns, through frequent opportunities for informal contact between clinicians that contributed to relationship building. Two non-UK qualitative studies examining barriers to SPC referral from the haematologists’ and palliative care specialists’ perspectives also show clear overlap with our findings, with difficulties identifying the end of life phase noted as a particular barrier to SPC referral [22, 26]. Interestingly, the strong patient-clinician relationship, perceived as reflective of quality care by some [47], was considered a barrier to the initiation of timely end of life discussions by others [26].

A growing body of international quantitative evidence now exists, particularly from the US, about the provision of palliative care for patients with haematological malignancies. These studies, conducted with haematologists and oncologists, also note concerns about the lack of blood transfusion provision within hospices and comment on inadequate awareness about the role of palliative care specialists [24, 48, 49]. More generally, a UK study including patients with other cancers also reported resistance to SPC and hospice referral due to the negative connotations of these services [50].

Perceptions of our interviewees about palliative care referral and the factors influencing this largely mirror those described in studies seeking to understand haematologists’ perspectives. This implies a degree of shared awareness across specialisms regarding the uniqueness of blood cancers and the issues differentiating patterns of care in these diseases from those of other cancers. It is likely that this mutual understanding will mediate future changes in practice that will facilitate improved patient care.

One strength of our study is that interviewees worked within differing SPC settings, thereby increasing the breadth of the findings. Closely matching reports from individuals working in different areas enhance the credibility of the results. Qualitative methods are suited to exploration of phenomena about which little is known [28] and the aim of purposeful sampling is to select ‘key informants’ who can provide rich description of the phenomena being studied. Our study sample yielded data that provide new insights into an important but under-researched area and sensitize readers to new ways of thinking. Representativeness is not usually a key aspiration in qualitative research, which has implications for the generalizability of findings. Instead of using the term ‘generalizability’, it is more useful to talk about the ‘transferability’ of findings in relation to their relevance for understanding similar issues and processes [31]. Extrapolation of findings should take into account any study-specific contextual factors (e.g. health-care infrastructure; universal health-care coverage etc.) that may limit transferability [51].

## Conclusion

A range of barriers and facilitators were identified that were said to influence the likelihood of SPC referral, some of which related to the organisation and delivery of SPC services, so were amenable to change, and others relating to the complex and unique characteristics and pathways of haematological cancers. Collaboration between haematology and palliative care specialists was considered beneficial and desirable, and was said to be increasing. Further research is needed to assess the extent to which palliative care is provided by haematology doctors and nurses and other generalists and ways in which uncertainty could be used as a trigger, rather than a barrier, to referral.

## Abbreviations

EPaCCS: Electronic Palliative Care Coordination Systems
GP: General Practitioner
HMRN: Haematological Malignancy Research Network
SPC: Specialist Palliative Care
UK: United Kingdom
US: United States
